# Keggin Structure, Quō Vādis?

**DOI:** 10.3389/fchem.2018.00346

**Published:** 2018-08-14

**Authors:** Aleksandar Kondinski, Tatjana N. Parac-Vogt

**Affiliations:** Laboratory of Bioinorganic Chemistry, Department of Chemistry, KU Leuven, Leuven, Belgium

**Keywords:** polyoxometalates, Keggin structure, structural chemistry, metal oxides, kegginoid, clathrates

## Abstract

Working under the supervisor of William Lawrence Bragg at the University of Manchester and being under the direct personal and scientific influence by Linus Pauling, Dr. James Fargher Keggin some 85 years ago published a highly unique discovery—the structure of phosphotungstic acid (Nature 1933, 131, 908–909). This structure sparked the reports of other related polyanions from Keggin's contemporaries, marking the true beginnings of structural polyoxometalate chemistry. In this perspective article, we unveil some aspects and applications of Keggin's structure and discuss how it has shaped the course of our understanding of polyoxometalate chemistry and nanomolecular metal oxides/hydroxides in general.

## Introduction

Over human history, many increments in the understanding of the properties of metal oxides have led directly to cultural and technological advancements (Brock, 1993; Arns, 1998; Sass, 2011). In the contemporary era of advanced materials (Heine, 2014), confinement of metal oxides/hydroxides to the nanoscale led to the emergence of cluster materials with unique physicochemical properties (Roy, 2011). Polyoxometalates (POMs) (Pope, 1983), also known as (hetero/iso)polyacids, are one of the earliest discovered metal-oxo nanoclusters with a documented scientific history of over two centuries (Baker and Glick, 1998; Gouzerh and Che, 2006). POMs are typically comprised of early transition metals in high oxidation states (mainly V, Mo, W, Nb, and Ta) and they have been attracting research attention due to their actual and potential applications in catalysis (Kozhevnikov, 2009; Wang and Yang, 2015), molecular magnetism (Gatteschi et al., 1993; Müller et al., 1998; Clemente-Juan et al., 2012) and life sciences (Rhule et al., 1998; Absillis et al., 2008; Bijelic and Rompel, 2015; Ly et al., 2015).

**Figure 1:**
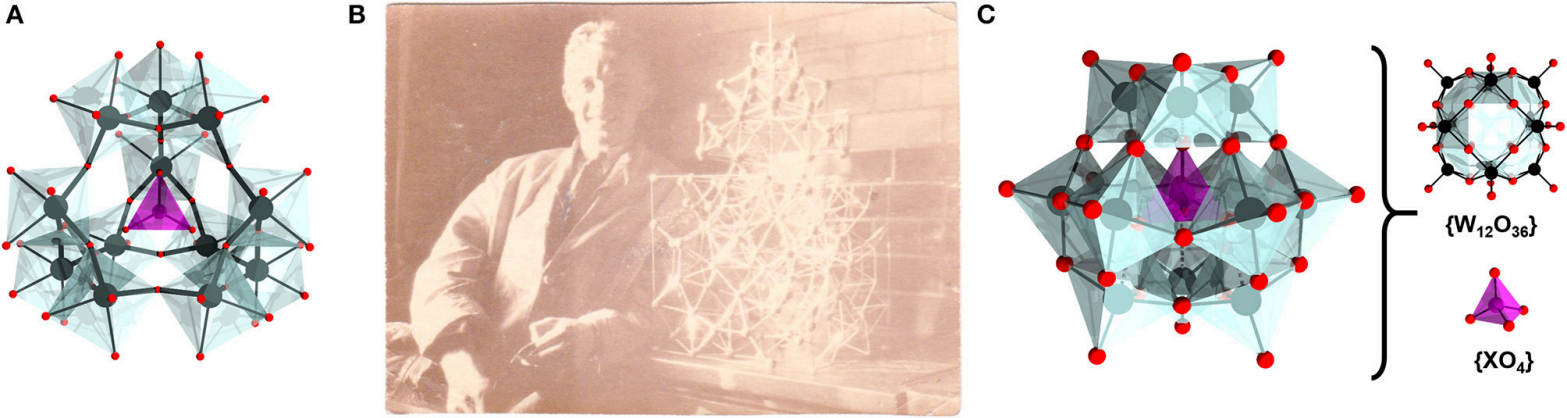
**(A)** Structural model of Pauling's {XO_4_}@{W(OH)_3_O_1.5_}_12_ model; **(B)** Photograph of Dr. James F. Keggin; **(C)** Keggin structure {(XO_4_)@M_12_O_36_} where M = Mo or W showing the different subunits. Color code: M, black; O, red; and X, purple.

Much of the modern POM chemistry is centered around the concept of the “Keggin structure,” which historically is representative for many heteropolymolybdates and heteropolytungstates. One of the earliest reports on these compounds dates back to 1826, when Berzelius reported the synthesis of phosphomolybdic acid (Berzelius, 1826). However, the structure of these species remained unknown for over a century. By late 1920's some of the renowned chemists working with POMs have attempted to apply Alfred Werner's coordination chemistry approach, which is based on covalent bonding between all constituents, to describe possible structural models (Baker and Glick, 1998). This has changed in 1928 when then a young professor Linus Pauling offered an unconventional solution to tackle the problem by introducing a structure (Figure 1A) that resembles what nowadays we refer to as a “host-guest” systems (Pauling, 1929a). Considering that Pauling's proposal preceded the foundation of modern supramolecular chemistry, at the time his model of the 12-heteropolyacids appeared as highly fascinating and controversial. In this model, a central {XO_4_} group is encapsulated by a neutral shell of {W_12_(OH)_36_O_18_} (also written as {W(OH)_3_O_1.5_}_12_). The shell of {W(OH)_3_O_1.5_}_12_ can be described as a virtual truncated tetrahedron constructed by twelve W and eighteen O centers defining the vertices and the edges respectively. Each of the twelve metal centers exhibits octahedral coordination and coordinates to three bridging oxo/oxido ligands and three terminal hydroxo/hydroxido ligands (Baker and Glick, 1998).

From the preserved written communications of Linus Pauling, it is clear that after the submission of his proposal (Pauling, 1929a) he contributed a significant effort to attract crystallographic interest to the problem (Pauling, 1928). As the X-ray crystallography was still in its early stage and very challenging to apply to large molecular systems, Pauling attempted to attract the interest of the Bragg's group at the University of Manchester, which at the time was globally the most prominent group on the powder crystal structure elucidation method. In 1930 Pauling visited Bragg's laboratory with the main purpose to discuss crystal structure elucidation of various compounds including the heteropolyacids (Pauling, 1929b).

During his visit at Manchester, Pauling met with Dr. James Fargher Keggin, which eventually sparked a turning point in the history of POM chemistry (Ayass and Kortz, 2018; Pütt et al., 2018). Dr. Keggin (31.12.1905–11.04.1993), born in the Isle of Man, was a physics graduate from University of Liverpool who performed synthetic and crystallographic experiments in Bragg's laboratory (Figure 1B). After the meeting with Pauling, Keggin's research focused on the study of the heteropolyacids. Within a couple of years, Keggin successfully resolved the positions of the tungsten and phosphorus atoms and estimated the relative position of oxygen atoms. Keggin's work was communicated in Nature in 1933 (Keggin, 1933a,b) while all details of the complete work appeared in 1934 (Keggin, 1934). Considering the complexity of the discovered structure, Keggin described the tungstophosphic acid as an overall tetrahedral species constituting of twelve {MO_6_} octahedra grouped in three triads of {M_3_O_15_} that connect in corner-sharing fashion around the single central {XO_4_} unit (Figure 1C). Over the decades, this topology became commonly referred to as the Keggin structure, while more recently in Keggin's honor, a naturally occurring POM mineral Pb_3_Ca_3_[AsV_12_O_40_(VO)]·20H_2_O with Keggin-like motif became known as *kegginite* (Kampf et al., 2017).

Within the next two decades, a handful of other structures have been proposed and structurally elucidated which laid the foundation of the modern field. However, besides the crystallographic elucidations, the attempts to predict structural topologies by some leading structural chemists at the time such as John Start Anderson (Imperial College London) and Alexander Frank Wells (University of Cambridge) are also very fascinating from the contemporary point of view. Anderson's proposal on the [IMo_6_O_24_]^5−^ structure representing a segment of typical double layer metal hydroxide M(OH)_2_ (Anderson, 1937), laid in general a correct prediction which was first confirmed for the [TeMo_6_O_24_]^6−^ analog (Evans, 1948). On the other hand, Wells' structural predictions were based on an approach that relates to the conceptual construction of polyhedral molecules such as metal-organic polyhedra (Tranchemontagne et al., 2008). Wells' proposal acknowledged the non-uniformity in chemical bonding among different molecular “aggregates” which include the heterogroup and the metal-oxo shell (Wells, 1940). In this regard, he proposed rational stepwise increase of the nuclearity of the molecular metal-oxo shells, while making it clear that [MO_3_]_n_ and their terminal-oxo free derivative [MO_2_]_n_ follow the same building up trend. In Wells' view, the Keggin structure can be described as a {M_12_O_36_} shell with cuboctahedral and rhombocuboctahedral networks defined by the positions of the M and O atoms respectively that hosts a heterogroup. Following the step wise building up, he envisioned that a [MO_3_]_18_ shell should provide sufficient room for two heterogroups leading to the {P_2_W_18_O_68_} structure. This structural proposal was confirmed a decade later by Barrie Dawson (Dawson, 1953; Mathieson, 1975).

When examining the {MO_6_} “octahedra” in the Mo^VI^ and W^VI^-based Keggin structures, the non-uniformity to which Wells was referring to becomes apparent. Although many detailed bonding studies exist (Mingos, 1999), one easily marks the large discrepancy in the M–O bond lengths which are ca. 1.7 and 1.9 Å from the addenda to the terminal and the bridging μ_2_-O atoms respectively, but ca. 2.4 Å from the addenda centers to the interior O atoms attached to the heterogroup. With the emergence of supramolecular chemistry, the non-uniformity in bonding gave rise to the clathrate-model in late 1980's and early 1990's (Pope and Müller, 1991), which later became a useful model for discussing the electronic properties of Keggin derivatives on the basis of computational chemistry (Maestre et al., 2001; Neiwert et al., 2002; López et al., 2006).

## Applications of the “classical” Keggin clathrates

The Keggin structure, as originally elucidated, is primarily representative for hetero-12-molybdates and hetero-12-tungstates. In this regard, multiple tetrahedral heterogroups {XO_4_} where X = P, Ge, Si, or As (but also many other different cations) have been reported to be encapsulated within the {M_12_O_36_} shell (Pope, 1983; Anyushin et al., 2014). The Mo^VI^- and W^VI^-based Keggin anions can be easily reduced forming blue and brown colored solutions respectively, due to the intense charge transfer processes. In this context, already starting from the nineteenth century there has been an application of this property in the colorimetric detection and quantification of phosphate anions by the so-called molybdenum blue method (Holman, 1943; Baker and Glick, 1998). Indeed, this redox-responsive and reversible change in color has made the Mo-based Keggin structure recently applicable for preparation of rewritable paper (Sun et al., 2015). The reduction of the Mo-based Keggin structure impressively undergoes up to 24 electrons process without disintegration of the structure (Wang et al., 2012), as the reduction also increases the formation of metal-metal bonding between the addenda centers (Nishimoto et al., 2014). It has been noted that the reversible redox processes of these polyanions are also very useful in the preparation of electrolytes suitable for light-driven artificial hydrogen evolution (Symes and Cronin, 2013). Typical Mo- and W-based Keggin structures exhibit thermal stability up ca. 300°C, making them catalytically attractive also at elevated temperatures (Moffat, 2002). As redox active polynuclear superacids they provide a reactive surface for a variety of (electro)catalytic transformations (Kozhevnikov, 2009). The high number of heavy elements and overall molecular surface covered by terminal oxo atoms makes the Keggin species also very suitable for applications in protein crystallography (Bijelic and Rompel, 2015).

The “Achilles heel” of the Mo- and W-based Keggin is that their stability is pH dependent. As the pH increases the Mo- and W-based Keggin gradually disintegrate. This property was one of the main challenges that in the early twentieth century made the assignment of overall charge to these polyanions very difficult as the resulting “unsaturated” or “lacunary” structures exhibit higher negative charges per addendum than the parent Keggin anion (Baker and Glick, 1998). However, later it was realized that the lacunary polytungstates encapsulating {PO_4_}, {SiO_4_}, {GeO_4_}, {AsO_3_}, {SbO_3_}, and {BiO_3_} heterogroups can be effectively isolated and used as polydentate all-inorganic ligands (Pope, 1983; Baker and Glick, 1998). Typically, the former Keggin-derived lacunary POMs include the monolacunary species {(XO_4_)W_11_O_35_} which are generated by a formal loss of single {MO} unit and a set of trilacunary heteropolytungstate conformers {(XO_4_)W_9_O_30_} and {(XO_3_)W_9_O_30_}, obtained by the loss of {MO_2_}_3_ unit. Such materials exhibit defect sites with available nucleophilic O centers that interact with any cation in solution which can be exemplified by their recent application in ultrahigh water purification of toxic metal cations (Herrmann et al., 2017). The lacunary W-based Keggins are typically prepared at higher pH (ca. 7–9) and have been employed as all-inorganic ligands in the preparation of many organometallic (Proust et al., 2012), lanthanide (Boskovic, 2017) and transition metal containing (Zheng and Yang, 2012) polyanions which show potential and actual applications in catalytic oxidations of organics (Proust et al., 2008), luminescence (Binnemans, 2009; Yamase, 2009), hydrolysis of biomolecules (Ly et al., 2015), stabilization of nanoparticles (Mitchell and de la Fuente, 2012), design of hybrid materials (Proust et al., 2008, 2012), smart surfactants (Landsmann et al., 2012; Polarz et al., 2014) molecular magnetism (Gatteschi et al., 1993; Clemente-Juan et al., 2012) and design of supercapacitors (Gómez-Romero et al., 2003).

## The “uniqueness” of the Keggin structure and the rise of molecular kegginoids

**Figure 2:**
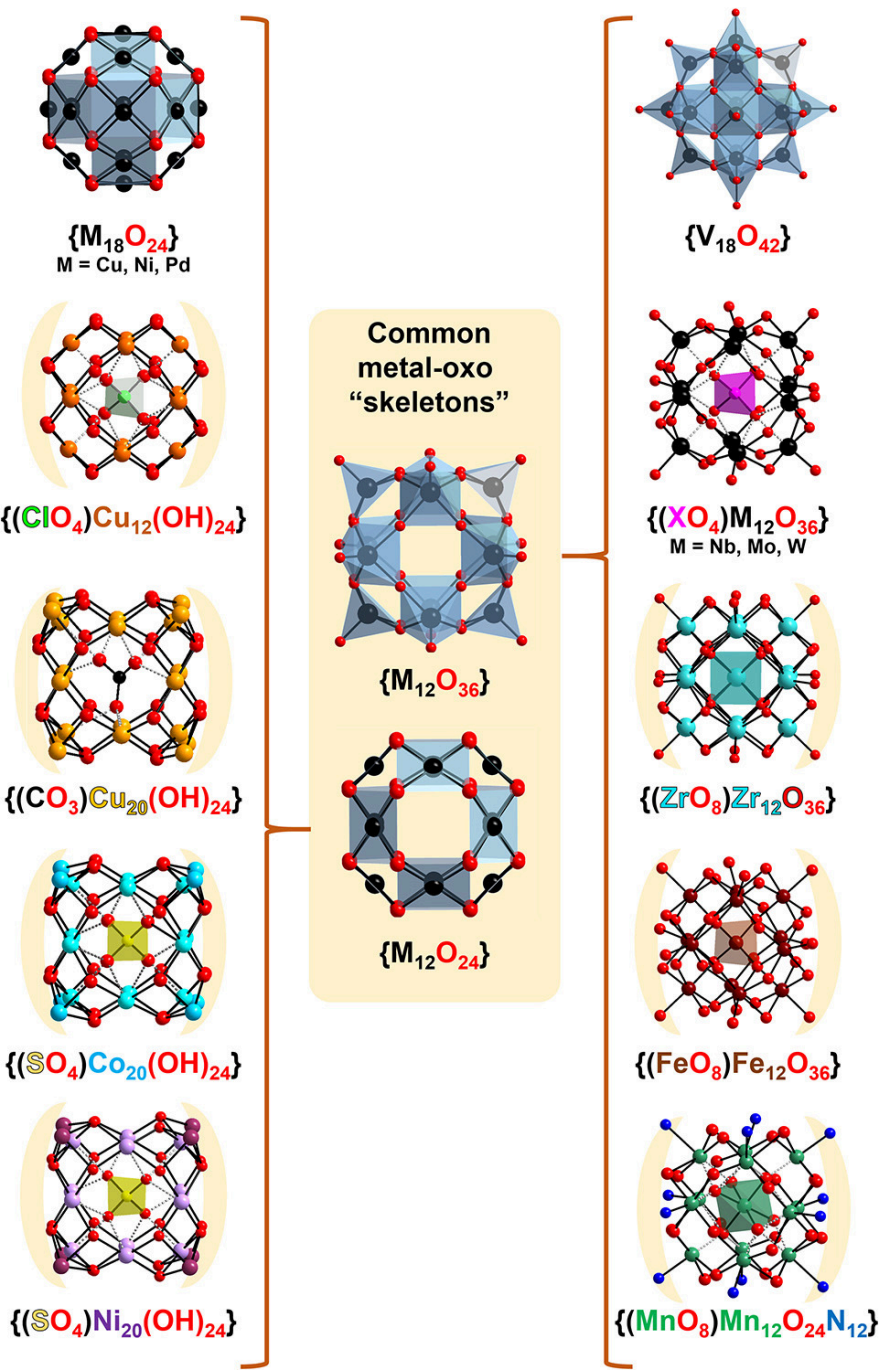
Ball-and-stick representation of the common {M_12_O_36_}/{M_12_O_24_} “skeletons” presented in the classical Keggin structure (Mo and W) and among the other transition metal based Kegginoids: V (Müller and Döring, 1991), Cu (Kondinski and Monakhov, 2017), Fe (Sadeghi et al., 2015), Co (Dong et al., 2013), Mn (Newton et al., 2011), Zr (Day et al., 2005) Ni, and Pd (Crooks and Weller, 1997; Dong et al., 2009).

Already from the mid-1960's to the end of 1970's it became well-established that hetetrometals (M') can substitute one or more addenda centers of the {M_12_O_36_} shell leading to mixed-addenda {(XO_4_)M'_x_M_12−x_O_36_} Keggin anions (Smith and Pope, 1973; Pope and Scully, 1975; Pope et al., 1976). These studies combined with the findings that other elements such as aluminum (Johansson, 1960) and germanium (Bradley et al., 1990) can form cationic assemblies similar to the Keggin structure, opened up the way for Baker-Figgis concept of Keggin configomers (Baker and Figgis, 1970), which implies that in principle the overall {M_12_O_36_} shell can adopt a variety of topologies that differ mainly on the ratio of edge-sharing vs. corner-sharing polyhedra. These studies were hinting that other elements may mimic POM topologies and in particular produce Keggin-like (Kondinski and Monakhov, 2017), “extended” Keggin (Müller and Döring, 1991) or as we herein refer to *kegginoidal* topologies (Figure 2). One of the early visions in this directions came from Müller and coworkers who recognized the relationship between “classical” Keggin structure and “extended” Keggin structure [X@V_18_O_42_]^n−^ (X = ${\text{SO}}_{4}^{2-}$ and ${\text{VO}}_{4}^{3-}$) (Müller and Döring, 1991). These structures indeed can be virtually derived by addition of {MO} cations at the unoccupied square faces of the virtual rhombocubochthedron constructed by the 24 bridging oxo ligands of the [MO_3_]_12_ shell. In the case of vanadium, the necessity of additional vanadyl cations is clear because the hypothetical fully-oxidized shell [V_12_O_36_]^12−^ is expected to be highly negative and thus highly reactive. Therefore, the virtual addition of vanadyl cations is supposed to charge-stabilize the overall structure. In the case of vanadium's heavier congener niobium, highly charged polyanions of the type [(XO_4_)Nb_12_O_36_]^16−^ (X = Si and Ge) (Nyman et al., 2004) exhibit very strong interactions with the counteractions in solution and in the solid state. Due to the high negative charge, the Nb-Keggin derivatives are also highly reactive with vanadyl cations producing kegginoidal [(PO_4_)Nb_12_O_36_(VO)_6_]^3−^ structure (Shen et al., 2014) representing mixed addenda derivatives of the {V_18_O_42_} structure.

However, besides recognizing the relationship between the classical and the “extended” Keggin shell system, in early 1990's Müller and coworkers also recognized the similarity between the kegginoidal {V_18_O_42_} and its polyoxocuprate analog that misses terminal oxo atoms [Cu_18_O_24_]^12−^ (see Figure 2; Kipka and Müller-Buschbaum, 1977). The kegginoidal topology of the former is also adopted by the polyoxopalladate [Pd_18_O_24_]^12−^ and the polyoxonickelate [Ni_18_O_24_]^12−^ (Crooks and Weller, 1997). These pioneering discoveries including number of reports of related heteropoly structures constitute the foundation of late transition metals based POMs (Ni, Pd, Pt, Cu, and Au) (Izarova et al., 2012; Kondinski and Monakhov, 2017) which exhibits potential in revealing insights into precious metal catalysis (Goloboy and Klemperer, 2009) and in building molecular nanomagnets with relevance to quantum computing (Baldoví and Kondinski, 2018).

The conceptual transitioning from {MO_3_}_12_ to {MO_2_}_12_ is an effective methodology that can assist in recognizing structural trends among a diversity of reported topologies. In this regard, copper(II) hydroxide which in the bulk shows characteristic chain structure (von Jaggi and Oswald, 1961), once confined to the nanoscale by stabilizing cations forms kegginoidal [Cu(OH)_2_]_12_ clusters (Kondinski and Monakhov, 2017). Normally, six or eight symmetrically arranged cations can stabilize the 24 labile hydroxo ligands. For instance, the naturally occurring [Cu(OH)_2_]_12_ kegginoid in the cavities of the zeolitic Tschörtnerite mineral is stabilized by eight Ca^2+^ cations (Effenberger et al., 1998). As synthetically it is challenging to mimic the environment of Tschörtnerite, many tenths of other synthetically reported kegginoidal polyoxocuprates exhibit metal-hydroxo cores where the local coordination of each individual copper(II) center besides square planar {CuO_4_} may adopt square pyramidal {CuO_5_} or octahedral {CuO_6_}, leading to large variety of structures with local differences, form which some to great extent mimic the classical Keggin (Kondinski and Monakhov, 2017).

There is a number of other structures that have been already recognized or that can be recognized as molecular kegginoids. For instance, the mineral murataite (Ercit and Hawthorne, 1995) exhibits kegginoidal {(ZnO_4_)@Ti_12_O_36_} motifs, which has been recognized and considered as a viable synthetic target (Nomiya et al., 2011). On the other hand, many polyalkoxotitanates resemble the structure of the {V_18_O_42_} kegginoid (Rozes and Sanchez, 2011). Titanium's heavier congener zirconium forms polyoxo/alkoxozirconate kegginoids which encapsulate a complete {ZrO_8_} unit (Morosin, 1977; Day et al., 2005). Nickel(II) and cobalt(II) based kegginoids [Ni_20_(OH_2_)_24_(MMT)_12_(SO_4_)]^2+^ (Dong et al., 2009) and [Co_20_(OH)_24_(MMT)_12_(SO_4_)]^2+^) (Dong et al., 2013; Mu et al., 2016) have been isolated in presence of MMT = 2-mercapto-5-methyl-1,3,4-tridiazole ligands. These polyoxocations exhibit {M_20_(OH)_24_} cores that can be derived by formal stabilization of the {M_12_(OH)_24_} topology by additional eight cations (Figure 2). In the case of manganese, mixed-valence kegginoid [${\text{Mn}}_{12}^{\text{III}}$Mn^IV^O_6_(OH)_2_(OMe)_4_(bemp)_6_]^4+^ (where H_3_bemp = 2,6-bis[N-(2-hydroxyethyl)iminometyl]-4-methylphenol) has been isolated and showed impressive solution and electrochemical stability and single molecule magnet behavior (Newton et al., 2011). Finally, among the most influential works on molecular kegginoids remains Nyman's “iron Keggin” [Fe^III^O_4_@Bi${}_{6}^{\text{III}}$${\text{Fe}}_{12}^{\text{III}}$O_12_(OH)_12_(Cl_3_CCOO)_12_]^+^ which has been successfully isolated and proposed as a good structural model of the naturally occurring ferrihydrite (Sadeghi et al., 2015).

All of these studies appearing over the past few decades require us to adopt a more inclusive vision of the Keggin structure and its role in the chemistry of nanomolecular metal oxides and hydroxides. In this light, the Keggin structure and its related molecular kegginoids unveil large opportunities for a conceptual approach to some practically important metal-oxo/hydroxo compounds. The conceptual approach proposes that structure-stability trends observed among Mo, W and V based kegginoids can be transferred and *in silico* explored for a variety of costly late transition noble metals. Such strategies could pave the way toward a rational synthesis of a variety of novel mixed-addenda, heterogroup substituted and even hybrid organic-inorganic kegginoids. Those approaches can also assist to shed light into the structure of some catalytically active metal oxides/hydroxides such as the Pearlman's catalyst (Pd(OH)_2_/C) (Albers et al., 2015).

## Conclusion and outlook

The discovery of the Keggin structure some 85 years ago was a turning point for structural POM chemistry. Over the past decades, global efforts have expanded the scope of applications of this structure, but more importantly, they have widened up our understanding of nanomolecular metal oxides. The structural similarities of the Mo- and W- based Keggin structure with many emerging metal-oxo/hydroxo/alkoxo clusters hint at the existence of common metal-oxo “skeletons,” which subsequently loosen the traditional boundaries of POM chemistry. So then where is the Keggin structure going? Considering that many metal cations may take a role as addenda centers or as stabilizing units, the landscape of molecular kegginoids that includes possible mixed-addenda, semimetal, and hybrid functionalized derivatives is limitless. Thus, with the ever-growing synthetic and theoretical insights into these systems, rational design and atom-economic preparation of novel kegginoids with desired properties are eminent.

## Author contributions

AK wrote the perspective. TP-V initiated and revised the perspective. Both authors have read and agreed with the final version of the perspective.

### Conflict of interest statement

The authors declare that the research was conducted in the absence of any commercial or financial relationships that could be construed as a potential conflict of interest.
